# Effects of Environmental Polycyclic Aromatic Hydrocarbons Exposure and Pro-Inflammatory Activity on Type 2 Diabetes Mellitus in US Adults

**DOI:** 10.4236/ojap.2022.112003

**Published:** 2022-06-24

**Authors:** Shweta Srivastava

**Affiliations:** Christina Lee Brown Envirome Institute, University of Louisville, Louisville, United States of America

**Keywords:** PAHs, Alcohol, CRP, HbA1C, T2DM

## Abstract

Polycyclic aromatic hydrocarbons (PAHs) are formed due to natural and anthropogenic activities and known for their potential impact and persistence in the environment. PAHs exposure has been linked to cause adverse health effect including lung cancer, heart conditions and genetic mutations. The understanding of metabolic effects of PAHs exposure is less clear especially in the presence of pro-inflammatory stress like alcoholism or diabetes. The aim of this article is to understand the metabolic effects of PAHs exposure on Type 2 Diabetes Mellitus (T2DM) by analyzing the clinical biomarkers data retrieved from the National Health and Nutrition Examination Survey, Center for Disease Control (CDC NHANES) (2015–16). This study has also accessed the interactive impact of PAHs and other proinflammatory factors, like alcohol intake on the metabolic syndrome on T2DM. We investigated urinary levels of hydroxylated PAHs metabolites (OH-PAHs) along with demographic, clinical and laboratory data. Generalize linear model Univariate factorial ANOVA was used to evaluate the group differences in the demographics, PAH exposure, drinking patterns, clinical data, and biomarker levels. Linear regression model was used to analyze the association of biomarkers, PAH exposure and drinking data. Multivariable regression model was used for multi-independent model to assess comorbidity association and their effect sizes on the clinical outcomes. The results indicated that BMI (p = 0.002), and age (≤0.001) are independent demographic risk factors for T2DM in high PAHs exposure. Acute proinflammatory activity characterized by CRP, is augmented by elevated monocyte levels (p ≤ 0.001) and stepwise addition of 1-Hydroxynapthelene (p = 0.005), and 2-Hydroxynapthelene (p = 0.001) independently. Prevalence of highest average drinks over time is observed in the high PAHs exposure; with males drinking almost twice compared to females in highly exposed population. Pathway response of T2DM shows sexual dimorphism; with males showing association with triglycerides (p ≤ 0.001), and females with CRP (p = 0.015) independently with HbA1C. The arrangement of CRP, absolute monocyte levels, serum triglycerides and average drinks over time predict the HbA1C levels (adjusted R^2^ = 0.226, p ≤ 0.001) in individuals with high PAHs exposure. Findings from this investigation support the pathological role of high exposure of PAHs in the exacerbation of metabolic disorder syndrome involving T2DM. Sexual dimorphism is reflected in alcohol drinking, with males drinking more in the high PAHs exposure group. Alcohol drinking as an independent factor was associated with the T2DM indicator, HbA1C in individuals with high PAHs exposure.

## 1. Introduction

PAHs are a group of chemicals and ubiquitous environmental pollutants linked to many adverse health effects [1] [2]. PAHs occur naturally due to incomplete combustion of carbon containing compounds in wood, coal, crude oil, and gasoline. Anthropogenic PAHs are produced from partial burning of motor vehicle exhaust, fumes from asphalt road, industrial exhaust and garbage [3] [4] [5]. PAHs are also an important class of compounds present in cigarette smoke, making it another major source of exposure [6] [7]. PAHs can also be present in food like charcoal grilled meat and processed food [8] [9]. National Institute for Occupational Safety and Health (NIOSH) recommends workplace air exposure levels of PAHs at less than 0.1 mg/m^3^ [10].

Upon entering the body, PAHs are metabolized and eliminated via urine. Urinary concentrations of PAHs metabolites, specifically mono-hydroxylated PAHs (OH-PAHs), have been used as biomarkers of human exposure to select PAHs, including naphthalene, fluorene, phenanthrene, and pyrene [11] [12].

PAHs form small airborne particles and cause a pronounced pulmonary inflammatory response including lung cancer [13] [14]. PAHs have also been associated with cardiotoxicity such as atherosclerosis, cardiac hypertrophy, arrhythmias, and contractile dysfunction [15] [16]. PAHs have shown to promote atherosclerosis and cause endothelial cell apoptosis [7] [17]. Liver is the major metabolic organ for PAHs, therefore transport of PAHs to systemic circulation significantly affects hepatic metabolism [18] [19] [20]. Inhaled PAH, Benzo[a]pyrene was demonstrated to metabolize and generate reactive oxygen species (ROS) in the liver, contributing directly to the hepatoxicity [21].

Metabolic syndrome is the accumulation of correlated metabolic risk factors including obesity, dyslipidemia, high blood pressure and insulin resistance [22]. Metabolic syndrome increases the risk of developing Type 2 Diabetes Mellitus (T2DM) [23]. PAHs have been found to increase the likelihood of T2DM by 78% – 124% [24] [25]. This risk is confounded by the fact that some PAHs increase independent risk factors of T2DM such as obesity, alcohol consumption, elevated blood pressure, and blood lipids are all increased by PAHs, indicating the metabolic syndrome disorder [26] [27] [28]. High levels of exposure to PAHs have been indicated to be positively associated with diabetes [29]. However, there are gaps in the understanding of the clinical presentation or altered mechanisms that can clearly ascertain the role of PAHs in T2DM. The adverse effects of PAHs on T2DM and its clinical marker, HbA1C are not well understood. Additionally, the association of PAH exposure with inflammatory markers such as CRP and contribution to metabolic syndrome is yet to be identified. Moreover, the cumulative effects of interaction of heavy or binge alcohol intake and PAHs exposure on the exacerbation of metabolic syndrome are not clear.

Majority of the research done so far are mainly focused on respiratory toxicity of inhaled PAHs leading to pulmonary adverse effects [30] [31]. Primary aim of this study was to characterize the exposure effect of PAHs on T2DM; and examine the modifying effects of alcohol intake, blood pro-inflammatory mediators and lipids. In the present study, the 2015–2016 NHANES data for seven urinary metabolites of PAH exposure namely (with their code in parenthesis for NHANES tracking): 1-hydroxynaphthalene (URXP01), 2-hydroxynaphthalene (URXP02), 2-hydroxyfluorene (URXP04), 3-hydroxyfluorene (URXP03), 1-hydroxyphenanthrene (URXP06), 2- & 3-hydroxyphenanthrene (URXP25), and 1-hydroxypyrene (URXP10) were used to assess their effects on T2DM. A model was also developed to describe the interaction of high PAHs with modifying risk factors on T2DM. Finally, the role of demographic confounders was also explored.

## 2. Methodology

### 2.1. Study Participants, Questionnaires, and Examination Data

Population data were taken from the 2015–2016 NHANES provided by the National Center for Health Statistics (NCHS) (https://wwwn.cdc.gov/Nchs/Nhanes/2015-2016/DEMO_I.htm). All the laboratory measurements were performed by CDC’s Division of Laboratory Sciences at the National Center for Environmental Health. Survey data were collected at the mobile examination center interview room on the day of the health exam by CDC. The demographics data extracted for the study includes information on the survey participant’s household interview and examination status, sex, age and pregnancy status. Examination data also included body measures and blood pressure. Questionnaire data collected the information for alcohol drinking history and known diabetes status.

### 2.2. Laboratory Data

All the laboratory methods for blood and urine samples were performed by NHANES that has been described thoroughly at: (https://wwwn.cdc.gov/nchs/nhanes/continuousnhanes/labmethods.aspx?BeginYear=2015). Laboratory measured data in the study are the levels of PAHs, Glycohemoglobin, Glucose, Cholesterol, Lipids, Triglyceride, complete blood count, Lymphocytes, Monocytes, and hepatitis conditions.

### 2.3. Analytical Method for Urinary PAH Measurement

The analytical procedure followed by NHANES, involved enzymatic hydrolysis of glucuronidated/sulfated OH-PAH metabolites in urine, extraction by on-line solid phase extraction, and separation and quantification using isotope dilution high performance liquid chromatography-tandem mass spectrometry (on-line SPE-HPLC-MS/MS) [11].

### 2.4. Selection of Eligible Participants

After data collection from the NHANES website, samples where data for any of the following was missing were excluded: missing examination status, age below 18 years, pregnancy, missing BMI, missing laboratory data for Glycohemoglobin or PAHs. Individuals with missing alcohol drinking data for the past 12 months were also excluded.

**Figure 1.**
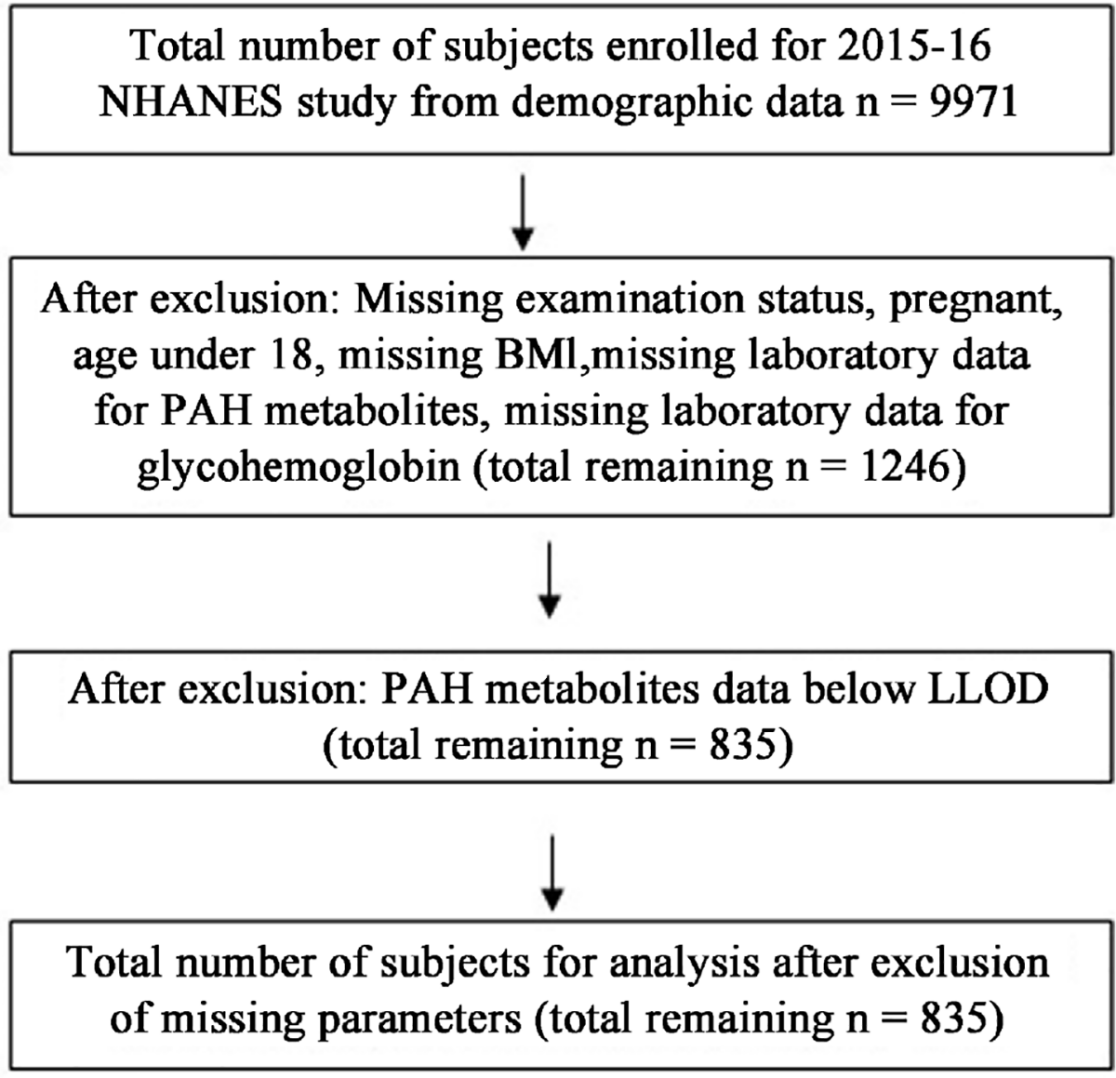
Flow Chart illustrating the selection of eligible participants. Eligible participants and those included in the analysis for the associations between urinary PAHs levels and other clinical parameters.

The specific urinary PAHs metabolites measured in this method are OH-PAHs, namely URXP01, URXP02, URXP03, URXP04, URXP06, URXP25, and URXP10 (In the NHANES database) (Figure 1).

### 2.5. Statistics

Participants were categorized for this investigation based on their PAHs metabolite exposure range as per NHANES data (Supplement Table 1). Populations based on each metabolite levels were categorized into tertiles. 1) below geometric mean exposure (>1990 ng/gm of creatinine), 2) geometric mean exposure (1990 – 2320 ng/gm of creatinine) and 3) higher than geometric mean exposure (>2320 ng/gm of creatinine) of the reference range for URXP01 calculated by NHANEs with 95% confidence interval (https://www.cdc.gov/exposurereport/pdf/FourthReport_UpdatedTables_Volume1_Jan2019-508.pdf). In this study, we utilized the exposure levels of 1-HN (stated as URXP01 in the NHANES database) as reference for the analyses purpose.

Male with >14 drinks/week, and females with >7 drinks/ week are heavy drinkers as per NIAAA classification (https://www.niaaa.nih.gov/alcohol-health/overview-alcohol-consumption/moderate-binge-drinking). Low risk or moderate drinking are who drank < 2 drinks/day as males, and females as <1 drinks/ day (https://www.cdc.gov/alcohol/fact-sheets/alcohol-use.htm). We used “alcohol drinks per day” over the past one year (ADPD) as our measure of interest for evaluating role of alcohol drinking.

Univariate factorial ANOVA was used to evaluate the group differences (both between the groups; as well as across all the groups) in the demographics, PAHs exposure, ADPD as measure of the alcohol drinking, clinical data, and biomarker levels for the study. Linear regression model was used to analyze the association of biomarkers and clinical, PAHs exposure and drinking data. Demographic data were used as covariates, if found to be significantly different in the between-group analyses. Multivariable regression model was used for multi-independent model to assess comorbidity association and their effect sizes on the clinical outcomes. IBM SPSS version 27 (IBM^®^, Armonk, New York USA) was used to compute all the statistical analysis and correlations in the study. Microsoft 365 version (Microsoft Corp., Redmond, Washington USA) was used for data processing and Table/s development. Figures were processed using GraphPad Prism (GraphPad, San Diego, CA USA). Data in table/s are presented as Mean and standard deviation (M±SD). Statistical significance was set at p < 0.

## 3. Results

### 3.1. Demographics, Drinking Profile and Diabetic Indexes by PAH Exposure Level

Age was significantly higher in Gr. 3 compared to Gr. 1 (Table 1). Notably, all the individuals in each group showed overweight to borderline obese, especially Gr. 1 and Gr. 2. Gr. 3 individuals drank on an average daily more than the rest of the two group individuals, and significantly higher (p = 0.015) compared to the Gr. 1 (Table 1).

### 3.2. Association of Diabetic Indexes and Demographic and Drinking Profile by PAH Exposure

**Figure 2.**
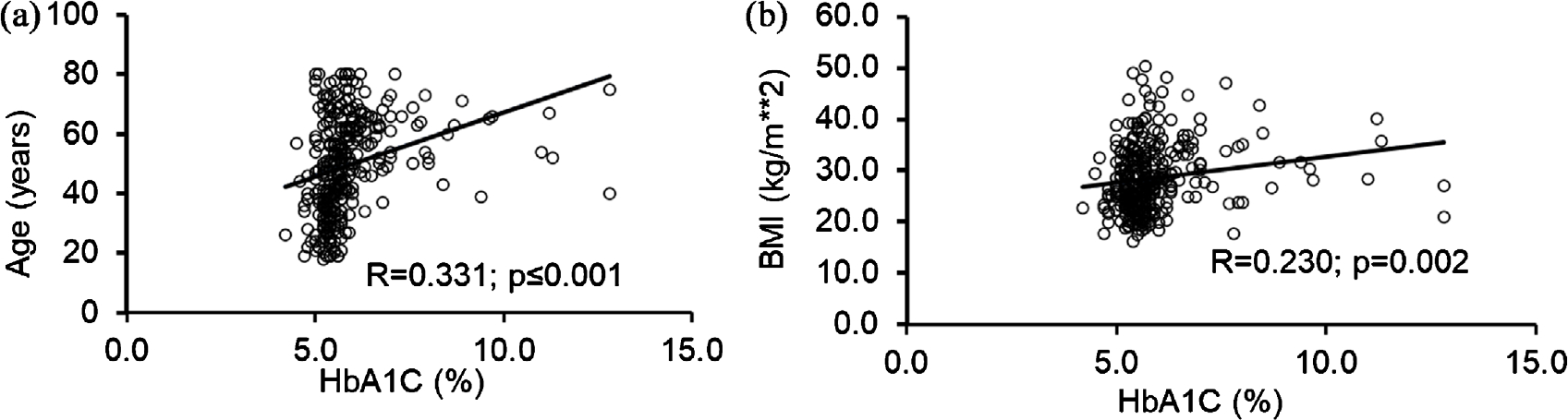
Association of HbA1C and demographic parameters in high exposure group 3 of the study. (a) Association of HbA1C and age. (b) Association of HbA1C and Body Mass Index (BMI). Raw data plotted on x- and y-axes. Effect sizes/correction index is noted along with the p-value. Statistical significance was set at p ≤ 0.05.

HbA1C and age were very significantly associated (R = 0.331, p ≤ 0.001) (Figure 2(a)) in Gr. 3 (high exposure group of 1-Hydroxynapthelene). HbA1C and BMI were also very significantly (p = 0.002) associated (R = 0.230) (Figure 2(b)) in Gr.3. In the univariate regression model, diabetic index (HbA1C) as a dependent variable showed unique relationship with candidate demographic indices exclusively in the high exposure group of 1-Hydroxynapthelene.

### 3.3. Association of Inflammatory Markers and PAH

**Figure 3.**
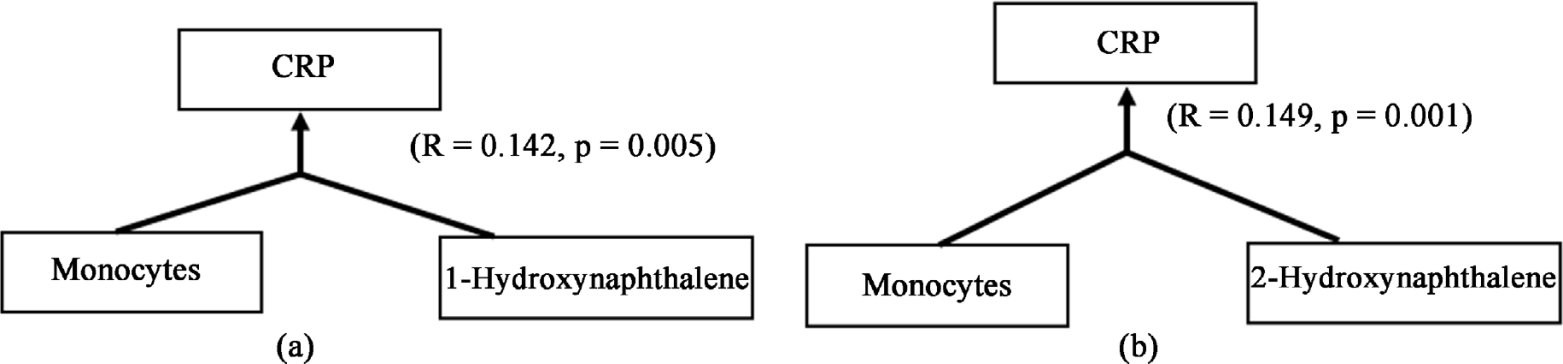
Association of C-Reactive Protein (CRP) with absolute monocyte count (AMC) in Group 3 of highly exposed subjects in multivariable regression model with PAHs. (a) 1-Hydroxynaphthalene; (b) 2-Hydroxynaphthalene. Effect sizes/correction index is noted along with the p-value. Statistical significance was set at p ≤ 0.05.

Univariate analysis of regression showed significant prediction (R = 0.141, p = 0.001) of C-Reactive Protein (CRP, marker of acute pro-inflammation) by the absolute Monocyte count levels (AMC) in the Gr. 3. Multivariable independent variable regression model was used to include the contributing role of candidate PAHs that could be potentially involved in the inflammation. CRP (as a dependent variable) showed augmented effects of association by AMC (as independent variable) and the candidate PAHs; 1-Hydroxynapthelene (R = 0.142, p = 0.005) (Figure 3(a)), 2-Hydroxynapthelene (R = 0.149, p = 0.001) (Figure 3(b)); respectively.

**Figure 4.**
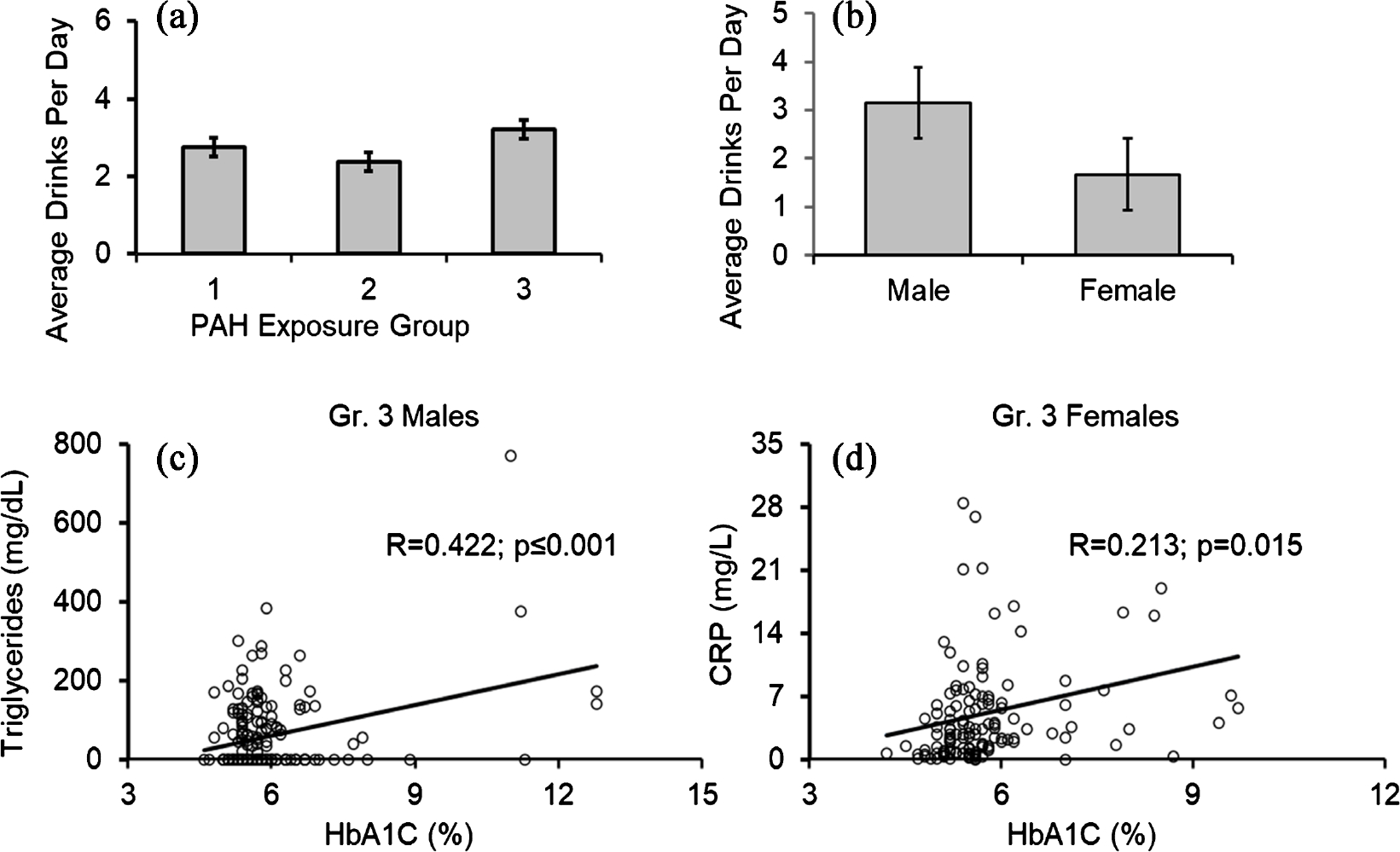
Alcohol drinking and PAHs exposure; and interaction of HbA1C (indicator of T2DM) and pro-inflammatory markers as sexual dimorphism in Gr. 3. (a) Gr. 3 individuals drank more than the other groups; (b) Males drank more than the females in Gr. 3; (c) Association of triglycerides and HbA1C in Gr. 3 males; (d) Association of CRP and HbA1C in Gr. 3 females. Group 1: below geometric mean exposure, group 2: geometric mean exposure and group 3: higher than geometric mean exposure of the reference range. Statistical significance was set at p < 0/05. Data presented as Mean ± Standard Error.

To estimate the role of alcohol drinking that is known to also cause inflammation; we reviewed the levels of ADPD in all the groups (Figure 4(a)). We found that Gr. 3 individuals have a propensity to drink more alcohol than the rest of the two groups. Noteworthy, in the Gr. 3, males drank significantly higher than the females (Figure 4(b)). Gr. 3 males also showed significant association of HbA1C and triglycerides (Figure 4(c)). On the other hand, Gr. 3 females showed significant association of HbA1C and CRP (Figure 4(d)).

**Figure 5.**
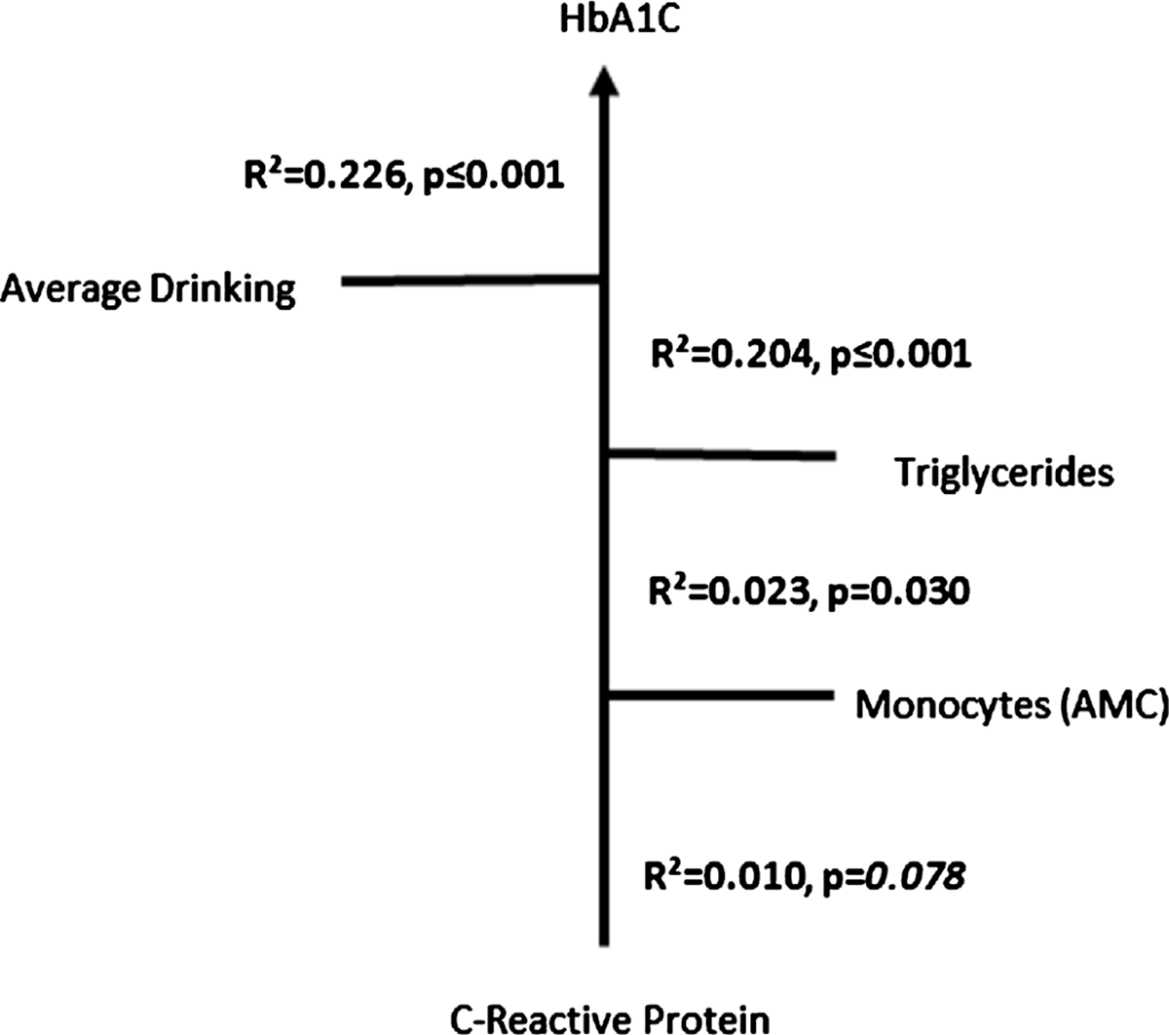
Stepwise multivariable regression analysis model illustrates the increasing effects on HbA1C as diabetic index by the pro-inflammatory activity presented as CRP, AMC, serum triglyceride along with average drinking per day in the Gr. 3. Statistical significance was set at p<0/05. R^2^ is noted corresponding with the p-values at each level of association.

With the findings on inflammation, immune activity and presence of alcohol; we evaluated the overall impact on the Diabetic index (HbA1C) in the context of CRP, candidate pro-inflammatory lipid/s, ADPD and immune activity in the Gr. 3 subjects. HbA1C was significantly predictable with CRP and AMC (Figure 5), although CRP by itself showed only a close to trend level of significance for its direct relationship with HbA1C. This arrangement further augmented its effects with simultaneously increasing order of statistical significance, when Triglycerides and ADPD were included in a stepwise manner as added independent variables in this multivariable prediction model.

## 4. Discussion

Findings from this investigation support the pathological role of high exposure of PAHs in the exacerbation of metabolic disorders, such as T2DM. Recent study on PAHs exposure in small population suggested elevation of inflammation (IL2, IL6, IL8) and oxidative stress marker (MDA) in prediabetic patients, indicating a metabolic syndrome enhanced oxidative damage [32]. Present study assessed the proinflammatory status and factors, risk factors and level of exposure to extend the present knowledge in a very large population data collected from NHANES. The analysis for the study was emphasized on 1-hydroxynaphthalene (URXP01) as assessment candidate and individuals exposed to this PAHs yielded some noteworthy results.

Age and BMI stood out as the demographic measures that showed propensity with T2DM diabetic index HbA1C in individuals with high PAH exposure of 1-hydroxynaphthalene, while individuals with normal or low level of exposure did not exhibit similar trend. Similar study performed on PAHs exposure and diabetes suggests a positive correlation among those two. However, no additive effect of PAHs and central obesity on diabetes was found [33].

Earlier studies have also reported association of inflammation markers and PAHs exposure [34] [35] [36]. We found similar assessments in the 2015–2015 dataset of NHANES database, especially in the context of the 1-hydroxynaphthalene, which was the scope of this study. CRP and inflammation associated blood cells (absolute monocyte count) showed a significant relationship in individuals with high exposure levels in this study.

One of the noteworthy observations in this study was that the individuals with high 1-hydroxynaphthalene exposure drank the most compared to the other exposure groups (normal/low). Males with high exposure of 1-hydroxynaphthalene had higher levels of average drinking than their female counterparts suggesting trends of sexual dimorphism. Almost a 2-fold higher average drinking in high PAHs exposed males might be uniquely worrisome for a possibly unidentified role of PAHs. Males having more average drinks is aligned with the findings of the National Alcohol Survey [37].

Earlier studies on mice have suggested that PAHs exposure induces lipid associated metabolic disorders in a time-dependent manner [38]. Similarly, CRP level observed in new-onset diabetes people diagnosed with HbA1c criterion has been recently reported [39]. However, there has always been a gap in how to address such findings in humans in context of PAHs exposure and other risk factors. In this study, males with high 1-hydroxynaphthalene exposure showed close association of HbA1C and triglycerides, suggesting sex-specific exposure associated response to metabolic conditions [40]. Females with high 1-hydroxynaphthalene exposure on the other hand showed that HbA1C was closely associated with pro-inflammatory activity (CRP), suggesting propensity to the risks of chronic conditions [41]. Such a sexual dimorphism in high PAHs exposure is a healthcare concern for metabolic disorder that needs more extensive evaluation; both mechanistically focus on biological pathways as well clinically with well-developed research questions.

In this study, several important clinical and biological measures involved in pro-inflammatory, pathological activity were observed to be higher as well as connected to each other. Thus, it was important to know their overall effect on the HbA1C levels in individuals with high PAHs exposure [32] [42]. Indeed, the effect size on the HbA1C levels grew in a stepwise manner in context of candidate markers with potential role in inflammation and injury. As described in the multivariable model, role of several markers (representing several pathways that can be affected) on HbA1C, support evaluating PAHs exposed individuals with comprehensive clinical assessment and varied tests to understand the complexity involved [43]. A study on a larger subject size such as this one, and its results may advance the understanding in the scientific literature on the adverse and harmful effects of PAHs exposure and its interaction with the risk factors that are attributed to the metabolic disorders.

None-the-less, this study has several limitations that also need to be discussed. This is a single timepoint observational study, and assessment of longitudinal data is not in the scope of this investigation. No biological mechanism was tested in an experimental model in this evaluation to validate the corresponding clinical observations. Thus, no causations/etiologies were identified or were the aims in this study. Risk factors, like BMI and age were identified in high exposure group of individuals. Aging and obesity associated with PAHs exposure could be the important newer directions of research that can offshoot from the findings of this study. The focus of this study was limited to one PAHs, 1-hydroxynaphthalene. However other PAHs are equally important to investigate and can also be evaluated as future directions as independent projects. This study also did not evaluate any other metabolic conditions.

In conclusion, this study identifies that BMI and age are likely to be candidate demographic risk factors for T2DM in individuals with high PAHs exposure. Acute proinflammatory activity characterized by CRP, an acute pro-inflammatory marker, is augmented when elevated monocyte levels are observed in relevance of and 1-hydroxynaphthalene; and 2-hydroxynaphthalene independently. Elevated CRP levels may also be predictive of development of the metabolic syndrome [44]. Prevalence of increased average drinks over time is also observed with high PAHs exposure, especially in males. With high PAHs exposure, T2DM shows sexual dimorphism. Males exposed with higher levels of PAHs show association with triglycerides, whereas females with CRP independently predicted HbA1C. Importantly, the levels of CRP, absolute monocyte levels (suggesting its activation), serum triglyceride levels, and average alcoholic drinks predict the HbA1C levels robustly in individuals with high PAHs exposure.

## Supplementary Material

1

## Figures and Tables

**Table 1.** Demographic, drinking, PAH, Lipids, blood cell values, C-reactive protein and diabetic markers are tabulated. Statistical significance was set at p ≤ 0.05. Data presented as Mean ± SD.

Measures	Group 1 (low) 1-Hydroxynaphthalene			Group 2 (Mid) 1-Hydroxynaphthalene			Group 3 (High) 1-Hydroxynaphthalene			*Across all groups p*-value

	Males n = 287 (56.8%)	Females n = 218 (43.2%)	Total n = 505 (100%)	Males n = 6 (46.2%)	Females n = 7 (53.8%)	Total n = 13 (100%)	Males n = 186 (58.7%)	Females n = 131 (41.3%)	Total n = 317 (100%)	

Age (years)^c^	45.57 ± 17.76	43.46 ± 17.58	44.66 ± 17.69	43.17 ± 22.41	44.71 ± 13.22	44.00 ± 17.24	49.94 ± 16.52	48.98 ± 15.88	49.54 ± 16.24	NS
BMI (kg/m^2^)^b#,c^	29.88 ± 6.33	31.507 ± 8.69	30.584 ± 7.48	29.23 ± 8.05	34.00 ± 7.37	31.80 ± 7.76	27.55 ± 5.66	29.83 ± 7.31	28.50 ± 6.48	≤0.001
Average Drink per Day in past 1 year	3.21 ± 2.71	2.09 ± 1.42	2.74 ± 2.32	3 ± 1.67	1.86 ± .69	2.38 ± 1.32	3.87 ± 2.80	2.21 ± 1.68	3.22 ± 2.65	≤0.037
**PAH metabolite level (ng/gm of creatinine)**										
1-hydroxynaphthalene (ng/g of creatinine)**	1164.49 ± 788.29	1098.06 ± 750.47	1135.81 ± 772.14	3726.81 ± 147.24	3760.83 ± 180.34	3745.13 ± 160.02	52465.91 ± 329582.65	165450.57 ± 845589.68	99156.73 ± 600710.65	NA
2-hydroxynaphthalene (ng/g of creatinine)^c^	5893.71 ± 7543.00	7866.85 ± 6303.15	6745.48 ± 7095.74	5561.87 ± 2065.07	8140.95 ± 2963.36	6950.61 ± 2821.07	13022.58 ± 8308.54	18245.61 ± 13760.66	15180.99 ± 11178.08	≤0.001
2-hydroxyfluorene (ng/g of creatinine)^a,b#,c^	234.74 ± 349.28	225.86 ± 279.47	230.91 ± 320.74	589.49 ± 226.21	336.84 ± 329.75	453.45 ± 304.75	1169.67 ± 1609.27	1184.38 ± 1138.77	1175.75 ± 1431.68	≤0.001
3-hydroxyfluorene (ng/g of creatinine)^a,b,c^	105.74 ± 116.42	95.19 ± 145.33	101.18 ± 129.66	279.80 ± 89.09	212.44 ± 260.72	243.53 ± 196.25	730.24 ± 761.15	786.40 ± 830.78	753.45 ± 789.87	≤0.001
1-hydroxyphenanthrene (ng/g of creatinine)^a#,c^	129.40 ± 123.75	135.78 ± 117.36	132.15 ± 120.96	286.67 ± 277.73	117.16 ± 58.92	195.40 ± 203.99	278.06 ± 528.98	278.04 ± 294.85	278.05 ± 446.74	≤0.001
2- & 3-hydroxyphenanthrene (ng/g of creatinine)^c^	168.42 ± 273.48	144.62 ± 93.45	158.15 ± 215.27	370.29 ± 401.85	153.63 ± 69.61	253.63 ± 286.96	473.01 ± 880.40	422.63 ± 614.16	452.19 ± 780.75	≤0.001
1-hydroxypyrene (ng/g of creatinine)^a,c^	137.14 ± 148.64	153.18 ± 100.39	144.07 ± 130.16	537.42 ± 693.53	131.80 ± 77.49	319.01 ± 497.71	419.58 ± 602.28	415.68 ± 461.92	417.97 ± 547.85	≤0.001
**Cholesterol and Triglycerides**										
Total Cholesterol (mg/dL)	188.11 ± 43.81	183.94 ± 38.79	186.33 ± 41.75	171.83 ± 54.52	193.86 ± 48.22	183.69 ± 50.31	187.70 ± 40.62	192.67 ± 39.51	189.76 ± 40.18	NS
HDL (mg/dL)^c^	47.79 ± 14.64	55.23 ± 16.92	50.96 ± 16.06	45.17 ± 11.50	61.71 ± 14.03	54.08 ± 15.07	51.45 ± 19.34	57.33 ± 16.62	53.88 ± 18.47	0.061^#^
LDL (mg/dL)^c^	112.30 ± 35.54	105.86 ± 30.42	109.59 ± 33.56	88.50 ± 72.83	132.67 ± 27.57	115.00 ± 47.87	115.67 ± 39.88	112.16 ± 32.13	114.36 ± 37.08	NS
Triglycerides (mg/dL)^a#^	106.16 ±63.16	99.82 ± 67.97	103.48 ±65.15	201.50 ± 118.09	132.33 ± 63.71	160.00 ± 83.37	129.01 ± 103.99	108.10 ±54.03	121.25 ± 89.12	0.045
**Candidate/Target Blood Cell Measures**										
Lymphocyte Absolute number (1000 cells/uL)^c^	2.07 ± 0.66	2.40 ± 0.85	2.21 ± 0.76	2.02 ± 0.47	2.09 ± 0.39	2.06 ± 0.41	2.23 ± 0.77	2.53 ± 0.75	2.35 ±0.77	0.015
Monocyte Absolute number (1000 cells/uL)^c^	0.60 ± 0.20	0.57 ± 0.18	0.59 ± 0.19	0.66 ± 0.38	0.54 ± 0.10	0.59 ± 0.25	0.63 ± 0.19	0.61 ± 0.20	0.62 ±0.19	0.071
**Blood Glucose and diabetes measures**										
Glycohemoglobin (%)^c#^	5.74 ± 1.07	5.71 ± 1.12	5.73 ± 1.09	5.45 ± 0.73	6.23 ± 1.75	5.87 ± 1.38	5.92 ± 1.16	5.78 ± 0.97	5.86 ± 1.09	NS
Glucose, refrigerated serum (mg/dL)	104.96 ± 43.22	99.57 ± 32.45	102.66 ± 39.06	108.17 ± 31.19	94.33 ± 12.08	101.25 ± 23.68	107.67 ± 47.28	101.98 ± 38.97	105.32 ± 44.06	NS
**Inflammation markers**										
HS C-Reactive Protein (mg/L)^c#^	2.93 ± 5.17	5.35 ± 7.08	3.96 ±6.17	5.58 ± 5.21	3.31 ± 5.41	4.45 ± 5.20	4.82 ± 10.72	5.30 ± 7.54	5.02 ± 9.53	NS

Footnote: BMI: Body Mass Index. HDL: High density Lipoprotein. LDL: Low Density Lipoprotein

a. Significant difference between group 1 and 2.

b. Significant difference between group 2 and 3.

c. Significant difference between group 1 and 3.

# Trend level of significance. a∼, b∼, c∼ #: Trend level of significant difference between two groups, respectively.

NS: Not Significant. NA: Not Applicable.

** Comparisons were not shown since the groups were distributed by the levels of 1-hydroxynapthalene, thus the differences were all significant.

## Abbreviations

ADPD: Average drinks per day past one year
AUD: Alcohol use disorder
BMI: Body mass index
CS: Clinically significant
HbA1C: Hemoglobin A1c or Glycated hemoglobin
HD: Heavy drinkers
HOMA: Homeostatic model assessment for Types 2 Diabetes
TChol: Total cholesterol
HDL: High density lipids
LDL: Low density lipids
MD: Moderate drinkers
PC: Polyaromatic Compounds
SEQN: Respondent sequence number
RIDSTATR: Interview/Examination status
RIAGENDR: Gender
RIDAGEYR: Age in years at screening
RIDRETH3: Race/Hispanic origin w/ NH Asian
RIDEXPRG: Pregnancy status at exam
BMXWT: Weight (kg)
BMXHT: Standing Height (cm)
BMXBMI: Body Mass Index (kg/m**2)
ALQ101: Had at least 12 alcohol drinks/1 yr.?
ALQ110: Had at least 12 alcohol drinks/lifetime?
ALQ120Q: How often drink alcohol over past 12 mos.
ALQ120U: # days drink alcohol per wk. mos. yr.
ALQ130: Avg # alcoholic drinks/day - past 12 mos.
ALQ141Q: # days have 4/5 drinks - past 12 mos.
ALQ141U: # days per week month year?
ALQ151: Ever have 4/5 or more drinks every day?
ALQ160: # days have 4/5 or more drinks in 2 hrs.
LBXGH: Glycohemoglobin (%)
T2DM: Type 2 diabetes Mellitus
URXP01: 1-Hydroxynaphthalene (ng/L)
URXP02: 2-Hydroxynaphthalene (ng/L)
URXP03: 3-Hydroxyfluorene (ng/L)
URXP04: 2-Hydroxyfluorene (ng/L)
URXP06: 1-Hydroxyphenanthrene (ng/L)
URXP10: 1-Hydroxypyrene (ng/L)
URXP25: 2 & 3-Hydroxyphenanthrene (ng/L)
LBXTC: Total Cholesterol (mg/dL)
LBDHDD: Direct HDL-Cholesterol (mg/dL)
LBXTR: Triglyceride (mg/dL)
LBDLDL: LDL-cholesterol (mg/dL)
LBXHSCRP: HS C-Reactive Protein (mg/L)
LBXSCR: Creatinine refrigerated serum (mg/dL)
LBXMOPCT: Monocyte percent (%).
