# Supplementary material for: Effects of Environmental Polycyclic Aromatic Hydrocarbons Exposure and Pro-Inflammatory Activity on Type 2 Diabetes Mellitus in US Adults
Source: Open J Air Pollut. Author manuscript; Available in PMC 2022 Jul 15. (PMC9283753; doi:10.4236/ojap.2022.112003)
Supplement: 1 [file NIHMS1821051-supplement-1.pdf]

Supplement Section

Table S1. Reference range table for urinary metabolites concentrations of polyaromatic hydrocarbons in males and females of US population normalized with creatinine value (Grainger, 2006).

Analyte	Geometric mean (95% confidence level) 2009-10~ or 2013-14~ year creatinine corrected in ng/gm of creatinine
	total
1-hydroxynaphthalene	2140 (1990-2320)
2-hydroxynaphthalene	3710 (3470-3970)
2-hydroxyfluorene	250 (237-264)
3-hydroxyfluorene	99.2 (93-106)
1-hydroxyphenanthrene	137 (132-143)
2- & 3-hydroxyphenanthrene	133 (127-140)
1-hydroxypyrene	125 (116-134)

Footnote: Derived from NHANES data Fourth National Report on Human Exposure to Environmental Chemicals Update (https://www.cdc.gov/exposurereport/pdf/FourthReport_UpdatedTables_Volume1_Jan2019-508.pdf).

Table S2. Names and source location of the data used from the NHANES data set.

Data type	Source	Variables
Demographic data	https://wwwn.cdc.gov/Nchs/Nhanes/2015-2016/DEMO_I.htm	Examination status, Age, Gender, Race, Weight and Pregnancy status
Examination data	https://wwwn.cdc.gov/nchs/nhanes/Search/DataPage.aspx?Component=Examination&CycleBeginYear=2015	Blood pressure and Body measures
Questionnaire data	https://wwwn.cdc.gov/nchs/nhanes/Search/DataPage.aspx?Component=Questionnaire&CycleBeginYear=2015	Alcohol use, Diabetes status
Laboratory data	https://wwwn.cdc.gov/nchs/nhanes/Search/DataPage.aspx?Component=Laboratory&CycleBeginYear=2015	Cholesterol, Triglycerides, Glycohoemoglobin, Hepatitis, Complete blood counts and PAH metabolites measurements

Grainger, J., Huang, W., Patterson Jr, D. G., Turner, W. E., Pirkle, J., Caudill, S. P., *et al.*, (2006). Reference range levels of polycyclic aromatic hydrocarbons in the US population by measurement of urinary monohydroxy metabolites. Environmental Research, 100(3), 394-423.
